# *Bougainvillea glabra* Choisy (Nyctinaginacea): review of phytochemistry and antimicrobial potential

**DOI:** 10.3389/fchem.2023.1276514

**Published:** 2023-10-19

**Authors:** Ingrid G. Ornelas García, Alma L. Guerrero Barrera, Francisco J. Avelar González, Norma A. Chávez Vela, Daniela Gutiérrez Montiel

**Affiliations:** ^1^ Centro de Ciencias Básicas, Laboratorio de Biología Celular y Tisular, Departamento de Morfología, Universidad Autónoma de Aguascalientes (UAA), Aguascalientes, Mexico; ^2^ Centro de Ciencias Básicas, Laboratorio de Estudios Ambientales, Departamento de Fisiología y Farmacología, Universidad Autónoma de Aguascalientes (UAA), Aguascalientes, Mexico; ^3^ Laboratorio de Biotecnología, Centro de Ciencias Básicas, Departamento Ingeniería Bioquímica, Aguascalientes, Mexico

**Keywords:** *Bougainvillea glabra*, bracts, traditional medicine, betalains, betacyanins, antimicrobial, phytochemistry

## Abstract

The *Bougainvillea glabra* or bougainvillea is a climbing plant native from South America belonging to the Nyctaginaceae family. The bougainvillea is recognized worldwide for its horticultural importance, due to the color of its bracts, commonly known as “flowers,” made up of bracts, which are the striking parts, and the true flowers, which are white and small. Bougainvillea is widely known in traditional medicine to treat respiratory diseases such as cough, asthma, and bronchitis, gastrointestinal diseases, also for its antibacterial and insecticidal capacity. The antimicrobial potential of the involucre of this plant has not been studied, despite research showing a high phytochemical presence of secondary metabolites such as alkanes, phenols, terpenes, and betalains. This review compiles information about the traditional uses of *B. glabra*, its botanical description, ecological relevance, phytochemistry, antimicrobial and antibiofilm activity, such as the toxicology of bracts and flowers.

## Introduction

Plants are part of the history of man, since antiquity they have served as a natural medicinal remedy to cure different diseases, the knowledge of these plants has been maintained from generation to generation by sorcerers, healers, or shamans (Azmir et al., 2013; Tugume and Nyakoojo, 2019).

The World Health Organization (WHO) reports that there are about 20,000 medicinal plants, which provide primary healthcare to more than 80% of the world’s population (Sasidharan et al., 2011; Tugume and Nyakoojo, 2019). For this reason, phytochemistry and pharmacology have used medicinal plants to investigate new ecological and biodegradable chemical entities that function in the treatment of different pathologies due to their central structures (Yusuf and Khan, 2022). In addition, the WHO recommends and promotes the use of herbal remedies in national healthcare programs, due to their low cost, popular acceptance, and safety by causing fewer side effects (Pandey and Tripathi, 2013).

Plant tissue produces secondary metabolites, which allow them to grow, reproduce, and defend themselves in stressful environments and are the main active principles of the plant which have biological activity with a variety of properties such as antimicrobial, anti-inflammatory, antioxidant, analgesic properties, among others; obtaining these active ingredients is achieved through extracts from different parts of the plant such as its leaves, stems, flowers, and fruits (Azmir et al., 2013; Abubakar and Haque, 2020; Che et al., 2021).

Infectious diseases caused by bacteria are one of the main health problems with high morbidity and mortality worldwide (Frieri et al., 2017). This is related to the resistance of bacteria to existing antibiotics, caused by the indiscriminate use of drugs, which is why it has been decided to obtain extracts from medicinal plants as possible antimicrobial agents (Dhankhar et al., 2013; Hemeg et al., 2020).

There are hundreds of plant species traditionally used as medicinal, but their active ingredients have not been fully studied, such as *B. glabra* Choisy, a climbing plant native to Brazil, belonging to the Nyctaginaceae family, which inhabit in warm climates, of great ornamental and horticultural importance, due to its striking “inflorescences”, formed by the involucre, which it is made up of a set of colorful bracts and the true flower (Tcherkez, 2004; Zahidul et al., 2016; Marrelli, 2021).

*B. glabra* is used in traditional medicine to treat respiratory diseases such as cold, flu, cough, bronchitis, and asthma, as well as for gastrointestinal problems such as diarrhea and dysentery (Schlaepfer and García, 2017; Rodríguez-Herrera et al., 2023). Properties with antimicrobial activity are also attributed to it due to the presence of active compounds such as flavonoids, tannins, alkaloids, phenols, betacyanins, terpenoids, glycosides, and essential oils (Edwin et al., 2007; Zahidul et al., 2016).

This review provides a comprehensive overview of botany, traditional uses, ecology, toxicology, phytochemistry, antimicrobial potential, and antibiofilm of *B. glabra* bracts and flowers, plant organs that are widely used in traditional medicine, but little investigated.

## Methodology

The research for information on *B. glabra* was carried out using different databases such as PubMed, Google Scholar, ResearchGate, eBook, Elsevier, as well as government and botanical pages. Information was included from 1994 to 2023. The research was carried out using the keywords “*Bougainvillea glabra*”, “buganvilia”, later Boolean operators were used such as: bracts and flowers, antimicrobial activity or biology, traditional medicine, phytochemistry and active principles; toxicity; botany and biology.

## Origin and distribution

The name of the genus *Bougainvillea* comes from the French naturalist and explorer Philibert Commerson, who discovered it for the first time in Rio de Janeiro, Brazil, in the year 1768, naming it in honor of his compatriot Louis Antoine de Bougainville, French explorer and navigator (Cumo, 2013; El-Sayed et al., 2021).

*Bougainvillea* is a plant of ornamental importance, endemic to South America. Pantropically introduced and distributed in warm regions of Mexico, Asia, Australia, the Caribbean, South Africa, the United States, and other countries (Lim, 2014).

## Botanical description of *Bougainvillea*

**FIGURE 1:**
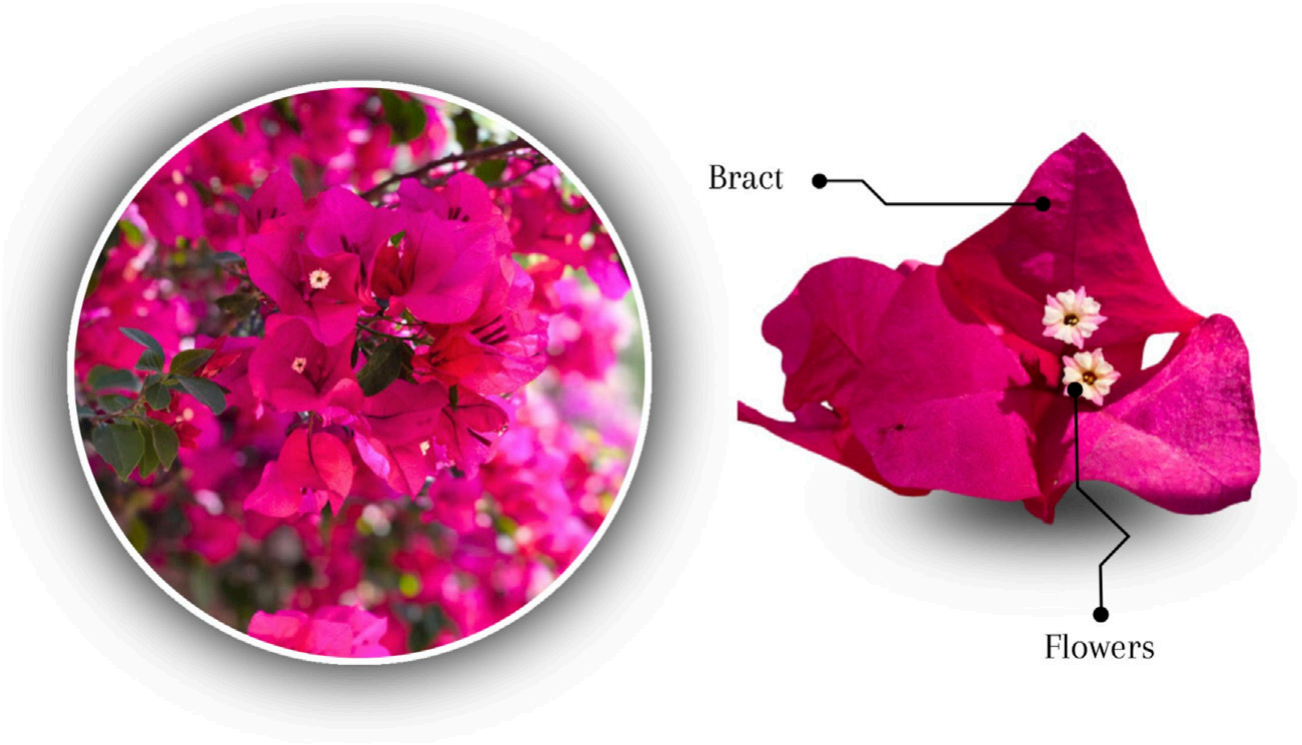
Bougainvillea: involucre constituted by bract and flowers.

They are woody or shrubby climbing plants; that present the leaf throughout the year. It has stems with thorns that help it climb; simple leaves, arranged alternately, entire, ovate to elliptic or lanceolate in shape; its flowers are small (Figure 1), tubular, appear in groups of three, white or yellow, bloom in spring and summer and even in early autumn; the flowers are surrounded by three colorful bracts, which have the consistency of paper, size and appearance of leaves; its fruit is elongated, no more than 1 cm long (Cumo, 2013; Napoleón et al., 2013).

Direct sunlight allows the growth and flowering of bougainvillea, as well as acidic and well-drained soils, with a pH of 5.5–6; they are tolerant to droughts (Napoleón et al., 2013).

## Taxonomy

The *Bougainvillea* genus belongs to the Nyctaginaceae family (Table 1), which houses around 33 genera and 400 species, from which Mexico reports 18 genera and approximately 110 species. *Bougainvillea spectabilis, B. glabra* and *B. peruviana* are the three most important horticultural species and the most studied. There are around more than 100 cultivars and hybrids that have not been studied (Gupta et al., 2009; He et al., 2020; El-Sayed et al., 2021).

**TABLE 1:** Taxonomic classification (Saleem et al., 2021).

Kingdom	Plant
Division	Magnoliophyta
Class	Magnoliopsida
Subclass	Caryophyllidae
Order	Caryophyllales
Family	Nyctaginaceae
Gender	Bougainvillea
Species	*Bougainvillea glabra* Choisy

## Botanical description of *Bougainvillea glabra*

**FIGURE 2:**
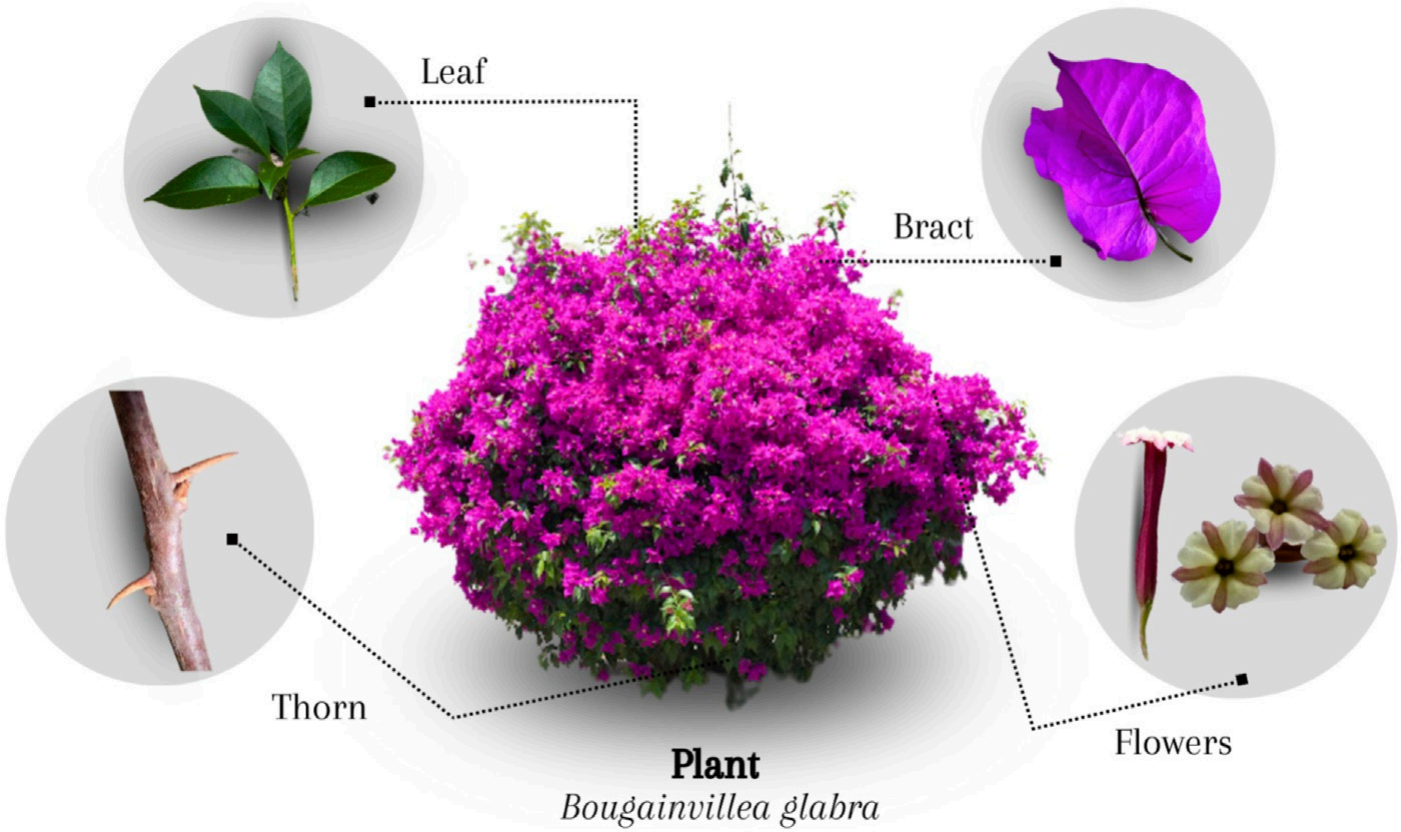
*Bougainvillea glabra* morphology.

Swiss botanist Jacques Denys Choisy identified *B. glabra* in 1850 (Napoleón et al., 2013). It is a perennial climbing shrub 1–7 m tall (Figure 2), with branches that have curved spines 5–15 mm long; simple leaves, dark green, somewhat glossy on the upper side, 1 cm long petiole, adaxially glabrous and abaxially pubescent, approximately 10 cm long; flowers 0.4 cm in diameter, bisexual, in a cymose inflorescence with three white to cream-colored flowers, perianth 1–2.5 cm long, slightly pubescent, with a single carpel, ovary and six to eight stamens; chartaceous bracts, ovate of 5 cm long and 1.54 cm wide, cardioid base and pointed tips, adhered to the flowers in the terminal region of the middle rib, of various colors; with small, dry, one-seeded and ribbed achene fruit. *B. glabra* habits warm, semi-warm, dry, semi-dry, and temperate climates (Lim, 2014; Saleem et al., 2021).

## Color of the bracts of *B. glabra*

The color of the bracts of *B. glabra* is due to the presence of pigments known as betalains (Napoleón et al., 2013).

**FIGURE 3:**
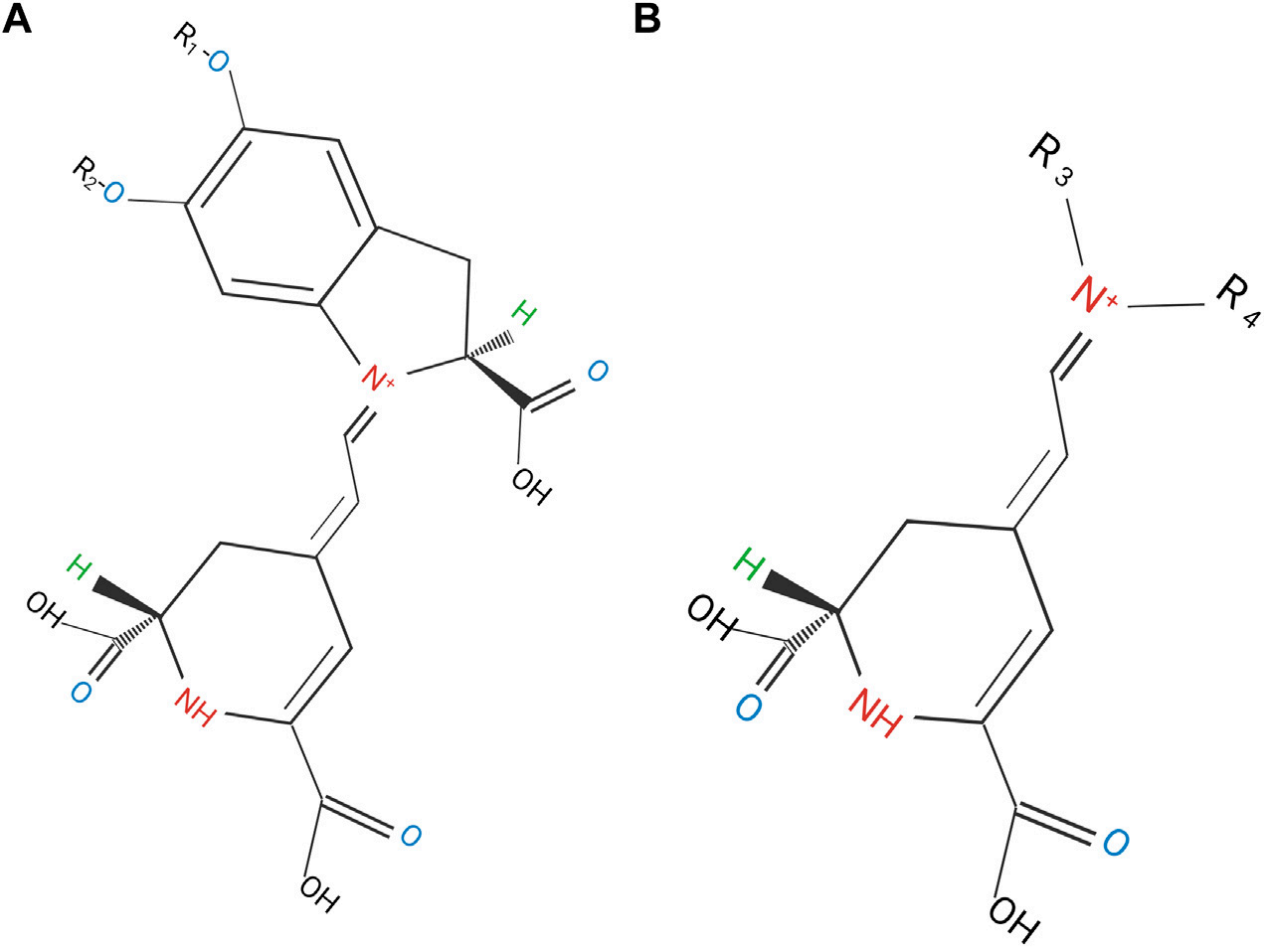
**(A)** Betacyanin structure: R1 and R2 represent Hydrogen or sugar moieties; **(B)** betaxanthin: R3 amino acid and R4: Hydrogen (Napoleón et al., 2013).

Betalains are water-soluble, vacuolar pigments, they present nitrogen with a heterocyclic ring in their structure. They are responsible for the color of the flowers, and fruits, as well as the leaves and roots of plants belonging to the Order Cariophyllales. Betalains are divided into betacyanins (Figure 3A) that are derivatives of betanidine, through an iminium adduct of cyclodioxyphenylalanine from the cyclo-dihydroxyphenylalanine (DOPA cycle), as well as betalamic acid, which provides a red-violet color; while the condensation of betalamic acid with α-amino acids or amines produce betaxanthins (Figure 3B) that provide a yellow-orange color (Vargas-Campos et al., 2018; Devadiga and Ahipa, 2020; Sadowska and Bartosz, 2021).

## Ecological importance

**FIGURE 4:**
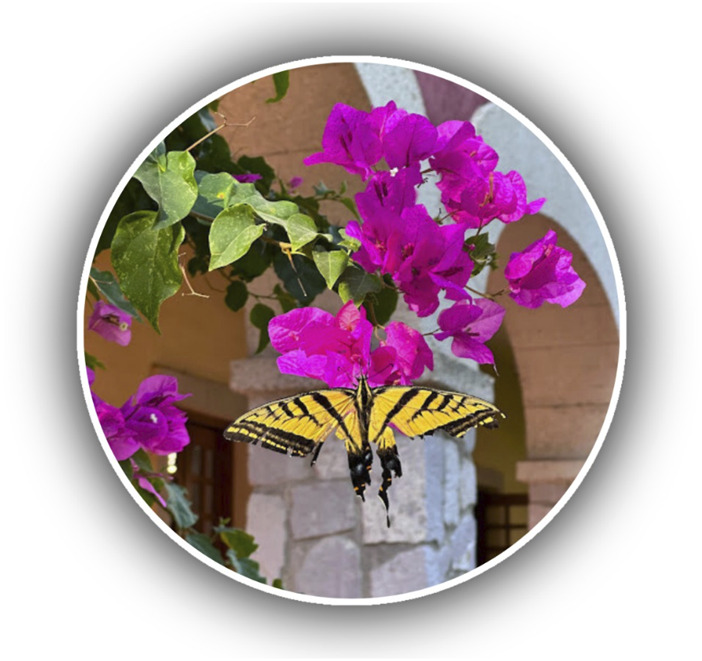
Bougainvillea being pollinated.

The coloration provided by betalains to the bracts of *B. glabra* favors the attraction of pollinators (Figure 4) and the dispersal of seeds (Sadowska and Bartosz, 2021). The attraction of pollinators is of utmost importance to our environment since they are responsible for 80% of the sexual reproduction of terrestrial plants, helping the functioning of ecosystems (Ghisbain et al., 2021).

Plants exposed to air-polluting particles have been reported to show tissue damage, changing epidermal cells and stomata. In a study carried out in India, leaves of *Bougainvillea* “Mahara”, *Terminalia arjuna*, *Cassia fistula*, and *Plyalthia longifolia* exposed to contaminating particles were collected, observing using a scanning electron microscope (SEM) that the bougainvillea did not present cuticular damage, indicating that this plant it acts as a mitigator of particulate pollution in urban and industrial areas (Kulshreshtha et al., 2009).

Therefore, the presence of plants such as *B. glabra* in urban areas is extremely important, not only to beautify the landscape but also to help mitigate the problem of pollution, as well as to maintain the functioning of ecosystems through pollination.

## Traditional uses and *importance* of *B. glabra*

**FIGURE 5:**
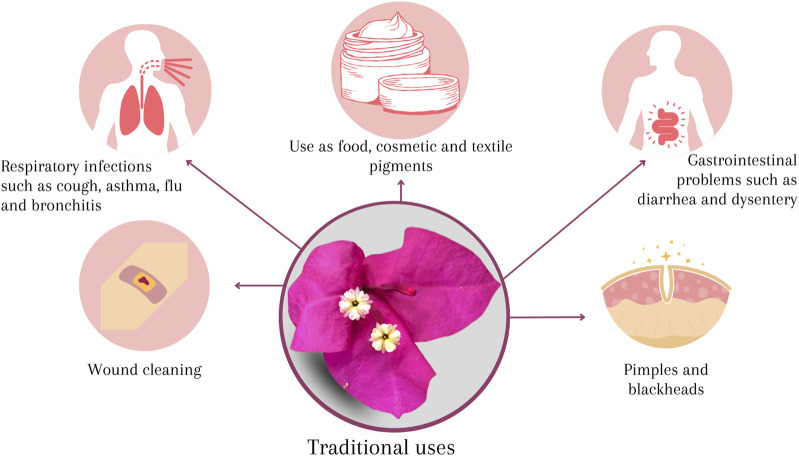
Traditional uses of *B. glabra*.

The involucre of *B. glabra* is widely used in traditional medicine to mainly treat respiratory diseases and different conditions (Figure 5).

In Mexico, *B. glabra* is popularly known by a wide variety of names such as purple bugambilia, paper flower, Santa Rita, but it is also named in indigenous languages such as shpupukuishonat (Mixtec), katsjoxhuan (Popolac) and jukua (Nahuatl) (Lim, 2014; Schlaepfer and García, 2017). The bougainvillea bracts, are commonly confused as flower petals, and these are the most used part in Mexican traditional medicine to treat respiratory conditions such as cough, asthma, flu and bronchitis through a variety of recipes; its use has also been reported to treat gastrointestinal problems such as diarrhea and dysentery; as well as to treat people who suffer from lung pain, whooping cough, drowning, urine sickness, pimples, and for cleaning wounds (Enciso-Díaz et al., 2012; Schlaepfer and García, 2017).

In Nigeria it is used to treat inflammation and as an analgesic (Ogunwande et al., 2019). In Thailand flowers are included in the daily diet to cure stomachache, and nausea (Kaisoon et al., 2012). In Mandsaur, India, bougainvillea helps reduce heartburn, treat sore throat, leucorrhea, blood vessels and hepatitis (Edwin et al., 2007; Gupta et al., 2009). To improve intestinal disorders extracts of *B. glabra* are used in Africa (He et al., 2020).

It has also been reported that extracts of *B. glabra*, popularly known as “glory of the garden”, work to increase collagen production, inhibit tyrosinase and TNF activity, and are recognized as antioxidant, antimicrobial, antiviral, insecticide, larvicide, antidiabetic, antilipidemic, antihyperglycemic, hepatoprotective, antiulcer, anthelmintic, antipyretic, antifertility, and anticancer (Ogunwande et al., 2019; El-Sayed et al., 2021; Saleem et al., 2021).

Other studies have focused on bougainvillea betalains to use them as food, cosmetic, textile and pharmaceutical pigments, due to their antioxidant and non-toxic properties (Kumar et al., 2017).

There are currently some natural bougainvillea-based syrups on the market to treat respiratory tract discomfort, but generally, these products are used only as supplements since there are no scientific studies that guarantee efficacy and safety (Enciso-Díaz et al., 2012).

In addition to its ethnobotanical application, *B. glabra* is classified as one of the plant species of great horticultural importance worldwide, due to its ramifications and abundant colorful inflorescences that create a surprising appearance on walls, gates, or pergolas in gardens (Cumo, 2013).

Despite the great variety of traditional uses, the study of the chemical and pharmacological properties of *B. glabra* is limited (Saleem et al., 2019).

## Phytochemistry of *Bougainvillea glabra* involucre

In 1970, the chemical components of the Bouganvillea genus began to be studied, using extracts from different organs of the plant (Abarca-Vargas et al., 2016). Next, the studies on the phytochemical composition (Supplementary Table S1) of the involucre of *B. glabra* are presented.

Due to its complexity, different techniques were used to determine the structure of the betacyanins present in the bougainvillea bracts. Using HPLC, about thirty complex patterns of betacyanins were detected. HPLC-MS-MS (Ultra-high performance liquid chromatography-MS/MS) recorded sixteen betacyanin precursor ions. In addition, nine structures were identified by HPLC- DAD (high performance liquid chromatography with a diode array detector), HPLC-MS and NMR-1D and 2D spectra, of which the latter helped to identify the betanidine fraction (Heuer et al., 1994).

In 2006, (Simon et al., 2006) using 1D and 2D nuclear magnetic spectroscopy (NMR) isolated three glycosides: momordin IIC (quinoside D), quercetin and a quercetin derivative, from an extract of the aerial part, without bracts, of *B. glabra*.

Using preparative ion-pair high-speed countercurrent chromatography coupled with electrospray ionization mass spectrometry (IP-HSCCC/ESI-MS-MS) six high molecular weight acyl-oligosaccharide-linked betacyanins were identified from a macerate of water, trifluoroacetic acid, and acetonitrile from bracts of a violet bougainvillea, collected in Guadalajara, Mexico (Jerz et al., 2010).

To know the phytochemical composition of four flowers used in the Thai diet: *Tagetes erecta*, *Cosmos sulphureus*, *Antigonon leptopus* and *B. glabra*; extractions with acidified methanol were performed and the compounds were identified by HPLC-DAD, where many phenolic acids and flavonoids were detected (Kaisoon et al., 2012).

Saleem et al. (2019) studied the phytochemical composition of methanol and dichloromethane extracts of *B. glabra* flowers using UHPLC-MS, revealing that most of the twenty-seven compounds are flavonoids and phenolic acids.

In Egypt, a study was carried out on the ‘Scarlett O'Hara variety bougainvillea, where the ethyl acetate fraction of the extract of the aerial part was used: stem, leaves and flowers, to detect different groups of metabolites using ultra-performance liquid chromatography with electrospray ionization quadrupole-linear ion trap tandem mass spectrometry, performed on ESI-MS positive and negative ion (UPLC-ESI-MS/MS), where about fifty-seven phytochemicals were detected, including seven organic acids, fourteen phenolic compounds, one betacyanin, seven anthocyanins, ten flavonoids, three saponins, six tannins, four cyclic tetrapyrolic derivatives and five miscellaneous (El-Sayed et al., 2021).

Knowing the variety of secondary metabolites present in the involucre of *B. glabra*, will allow us to improve its biological application in the future since it has been reported that the extracts that contain betalains present a variety of activities such as the inhibition of the growth of bacteria, as well as the yeasts and molds also prevent virus replication and have been reported to limit the growth of parasites. In the United States, betalains are patented as components of anticancer drugs due to their low cytotoxicity. On the other hand, it has been documented that they help reduce dyslipidemia, diabetes and have hepatoprotective, anti-inflammatory, neuroprotective and cardiovascular effects. These properties have been reported in clinical trials that provide safety to the use of these compounds, but they have only been studied for the genus *Opuntia* and red beet (*Beta vulgaris*), so the presence of these compounds in extracts of *B. glabra* opens a new opportunity to obtain and apply it (Devadiga and Ahipa, 2020; Sadowska and Bartosz, 2021).

Polyphenols are the most diverse group of secondary metabolites present in plants, more than 8,000 structures are currently known, and they are classified into phenolic acids, flavonoids, lignans, stilbenes, and tannins; to observe a variety of these compounds in extracts of *B. glabra*, which would benefit their study of new drugs since they have been reported to provide a wide variety of biological activities: they are natural anticancers due to their antioxidant and anti-inflammatory properties; they reduce the progress of neurodegenerative and cardiovascular diseases, they are excellent antithrombotic, antiallergic, anti-inflammatory and antimicrobial agents (Gorzynik-Debicka et al., 2018; Lobiuc et al., 2023).

In addition, it is important that when characterizing an extract, seasonal, local, and ontogenetic variations are reported when collecting the species, since this influences the phytochemical profile of the plant and therefore the pharmacological response (Enciso-Díaz et al., 2012). A challenge that is observed in the phytochemical studies of *B. glabra* is the correct identification of the plant organ, in addition, the choice of the color of the bract would offer a better identification between the pigments betacyanin and betaxanthin.

The presence of betacyanin in *B. glabra* has also been detected by Fourier-transformed infrared spectroscopy (FTIR), observing different absorption bands characteristic of the following functional groups (Table 2).

**TABLE 2:** Functional groups of betacyanins detected by FTIR.

Functional group	Absorption band (cm^-1^)	References
OH	3394	Kumar et al. (2017); Pérez et al. (2017)
	2527	
COOO-H	3281	
N-H		
CH	2953	
	2914	
	2842	
CΞC	2148	
C=O	1781	
	1651	
C=C	1451	
	1412	
	821	
C-O	1115	
	1040	
	1018	
N-H	1564	
	718	
COO-	1517	
C-N	1362	
C-OH	1272	
	1040	
OC-OH	880	

## Antimicrobial activity

Microorganisms are the major contributors to mortality worldwide due to infectious diseases. Currently the proliferation of diseases caused by pathogenic microorganisms is a risk factor for public health, these diseases are prevented by antibiotics, but due to their scarcity and current resistance of microorganisms, the use of phytochemical compounds from plants has been chosen for its medicinal properties due to their antimicrobial functions (Napoleón et al., 2013; Saeloh and Visutthi, 2021).

In recent years, it has been decided to scientifically investigate the use of *B. glabra* against bacteria and fungi that induce respiratory conditions (Table 3), based on the traditional use of involucre.

**TABLE 3:** Antimicrobial activity of bougainvillea wrapper.

Plant part	Extract	Inhibited microorganisms	Dose	Inhibition zone (mm)	References
Flower	96% ethyl alcohol.	*Staphylococcus aureus*	NA	9.4	Cardona et al. (2017)
		*Pseudomonas aeruginosa*		9.85	
		*Escherichia coli*		8	
Flower	95% methanol with successive extractions of n-hexane, carbon tetrachloride and water.	*Staphylococcus aureus*	20 mg/mL	17–22	Zahidul et al. (2016)
		*Escherichia coli*		15–16	
		*Bacillus cereus*		12–14	
Bract	Methanol-aqueous	*Bacillus subtilis*	60 µL	7.4	Napoleón et al. (2013)
		*Pseudomonas aeruginosa*		5.3	
Flower	95% ethanol	*Pseudomonas aeruginosa*	NA	7	Perales and Leysa (2012)
		*Escherichia coli*		7	
Part area	Ethanol and water (90:10) with differential extracts of hexane, ethyl acetate, and butanol	*Coccidioides immitis*	NA	CIM = 500 μg/mL	Alanís-Garza et al. (2007)

Perales et al. (Perales and Leysa, 2012) made 95% ethanolic extracts of leaves, stems, roots and flowers of *B. glabra* to test their antimicrobial activity against two Gram-positive bacteria *Bacillus subtilis* and *Staphylococcus aureus*, as well as two Gram-negative bacteria *Escherichia coli* and *Pseudomonas aeruginosa* using the Kirby-Bauer diffusion method, as a positive control, amikacin 30 mcg, cephalexin 30 mcg, oxacillin 1 mcg and penicillin 10 U were used, and distilled water as a negative control. The flower extract was effective only against Gram-negative bacteria *E. coli* and *P. aeruginosa*, with a zone of inhibition of 7 mm, respectively. It is ensured that the extracts can have a greater action on Gram-negative bacteria because they do not have an external membrane (Lobiuc et al., 2023).

The antioxidant and antibacterial activity of betacyanins from *B. glabra* bracts was investigated by aqueous-methanol extraction. The antimicrobial activity was evaluated using the well diffusion technique against the bacteria *B. subtilis, P. aeruginosa* and *E. coli*, using Ampicillin as a control. The betacyanins showed greater antibacterial activity against *B. subtilis*, inhibiting a zone of 6.7–7.4 mm, against *P. aeruginosa* they inhibited 4.8–5.3 mm, and for *E. coli* only from 3.4 to 3.7 mm (Napoleón et al., 2013). The antimicrobial action of betalains, like betacyanins, has been reported to mainly affect the structure and permeability of the cell membrane (Sadowska and Bartosz, 2021).

To evaluate the antimicrobial and antioxidant activity of the *B. glabra* flower, (Zahidul et al., 2016) carried out a flower extraction with 95% methanol for 2 weeks at room temperature, later fractions of the extract were subjected to successive extractions of n-Hexane and carbon tetrachloride. The antimicrobial activity was evaluated against *S. aureus, B. cereus, P. aeruginosa* and *E. coli* with the disk diffusion method, counting the antibiotic Imipenem as a positive control and each solvent as a negative control. The bacterium that presented the greatest zone of inhibition was *S. aureus* (17–22 mm), followed by *E. coli* (15–16 mm), *B. cereus* (12–14 mm), while *P. aeruginosa* showed the greatest sensitivity low (0–6 mm). A preliminary phytochemical analysis of the extract demonstrated the presence of alkaloid, flavonoid, tannin, phenolic compound, reducing sugar, amino acid, and protein. The antimicrobial activity of the extract may be due to the presence of hydrophobic flavonoid that penetrate the nonpolar core of the bacterial cell membrane, or hydrophilic flavonoids that form hydrogen bonds with the polar groups of membrane lipids; furthermore, the presence of quercetin causes DNA breakage and inhibits bacterial gyrase. The presence of tannin prevents bacterial growth by chelating iron and prevents cell wall synthesis by inactivating enzymes. While phenolic acids damage the cell membrane of Gram-positive bacteria, and the cytoplasm of Gram-negative ones; gallic acid alters the hydrophobicity, charge, and permeability of the membrane (Lobiuc et al., 2023). On the other hand, saponin causes the release of proteins and enzymes and alkaloids interfere with cell division (Hemeg et al., 2020)

Cardona et al. (2017) prepared infusions of leaves and flowers of *B. glabra* in ethyl alcohol at a concentration of 96%, which were kept for 2 months in the refrigerator. To measure the antibacterial activity, the disc diffusion method (Kirby-Bauer technique) was used against strains of *S. aureus, P. aeruginosa* and *E. coli*; each susceptibility test had a control to rule out that 96% ethyl alcohol had antibacterial activity. The results obtained showed that the leaf extracts have a higher inhibition against the strains of *S. aureus* (15.4 mm) and *P. aeruginosa* (16.8 mm); while flower infusions inhibited only 9.4 mm against *S. aureus*, and 9.8 against *P. aeruginosa*. An explanation for the fact that leaf extract presented greater inhibition could be due to its difference from the flower in its chemical composition.

The antifungal activity of 15 plants from northeastern Mexico against the fungi *Candida albicans, Aspergillus fumigatus, Histoplasma capsulatum* and *Coccidioides immitis*, inducers of pulmonary mycosis, has also been evaluated. The extraction of the aerial part of the plants was carried out with ethanol and water (90:10), subsequently, those extracts which presented antifungal activity were subjected to differential extracts of hexane, ethyl acetate and butanol. *B. glabra* only showed antifungal activity against *C. immitis* with a minimum inhibitory concentration (MIC) of 500 μg/mL of the hydroalcoholic extract (Alanís-Garza et al., 2007).

Although there is only one study that reports the minimum inhibitory concentration (MIC) of the *B. glabra* extract, in Supplementary Table S1 we observe that the different phytochemicals isolated from this plant present antimicrobial activity (MIC, minimum bactericidal concentration MBC, and half maximal inhibitory concentration IC_50_) against a wide variety of microorganisms (Shi et al., 2016; Wang et al., 2019; Taheri et al., 2020) of clinical importance, such as the pathogens known as ESKAPE, which include *Enterococcus faecium*, *S. aureus*, *Klebsiella pneumoniae*, *Acinetobacter baumannii*, *P. aeruginosa* and *Enterobacter* species that are highly infectious and resistant to multiple drugs, which are also classified by the WHO as high priority pathogens (*E. faecium* and *S. aureus*) and critical priority (*A. baumannii*, *P. aeruginosa* and *E. coli*) for the search for new drugs (Wijesinghe and Choo, 2022).

Therefore, the great variety of secondary metabolites present in the natural extracts makes it possible to inhibit or retard bacterial growth using different mechanisms of action, but due to the diversity of both compounds and bacteria, these mechanisms are still not well understood. It has been reported that the activity differs according to cell morphology, with coccoid cells being more resistant than rod cells (Nazzaro et al., 2013; Lagha et al., 2019).

## Antibiofilm activity

In recent years it has been reported that under natural conditions bacteria commonly live in biofilms, instead of their planktonic form (Rabin et al., 2015; Stiefel et al., 2016). Biofilms are bacterial communities covered by an extracellular matrix composed of extracellular polymeric substances (EPS), such as polysaccharides, proteins, lipids, and extracellular DNA, that adhere to surfaces (Cao et al., 2020; Furner-Pardoe et al., 2020).

The presence of EPS prevents the removal of biofilms from requiring up to 1000 times higher concentrations of antibiotics than planktonically grown bacteria (Stiefel et al., 2016; Furner-Pardoe et al., 2020), which causes around two million illnesses and more than 23,000 deaths per year due to resistant bacteria (Cao et al., 2020).

The high rate of resistance of biofilms to antibiotics is still not very clear, but the search for different and new antimicrobial agents has been chosen, such as the use of plant extracts, antimicrobial nanoparticles, antimicrobial proteins, and peptides (AMP), as well as antimicrobial enzymes (Cao et al., 2020).

Studies show that the use of secondary metabolites has antibiofilm activity since they alter the structure of the biofilm causing bacterial detachment, they also inhibit its adherence and affect the morphology of the biofilm (Roy et al., 2018).

The antibiofilm activity of flavonoids has been reported, which initially allows bacterial aggregation by membrane fusion, but then reduces the absorption of active nutrients, causing their death; in addition, they interact with sortases enzymes of the cytoplasmic membrane of Gram-positive bacteria, catalyzing the assembly of cells that allow infection. On the other hand, the inhibition of the expression and activity of the urease gene by the action of tannic acid reduces the formation of biofilms (Gorzynik-Debicka et al., 2018). A polyphenol extract manages to block the activity of glycosyltransferase (GTF) which affects the formation of the *S. mutans* biofilm (Cao et al., 2020). The reduction in the expression of virulence genes because of eugenol prevents the adhesion and formation of biofilms (Roy et al., 2018).

The antibiofilm activity of *B. glabra* extracts has not been explored, despite its potential due to the presence of a variety of metabolites, but a study carried out by Rauf et al. (2019) where zinc oxide nanomaterials (ZnO-NMs) were synthesized from aqueous extract of Bougainvillea sp. flowers, demonstrated their inhibitory effect on the development of *S. aureus* and *E. coli* biofilms at a concentration of 100 μg/mL for 48 h. Cao et al. (2020) mention that ZnO-NPs have antimicrobial activity by producing reactive oxygen species (ROS) that cause cell death and alter the stability of the cell membrane; they further hinder the EPS of biofilms and bind and inhibit DNA and enzymes.

## Toxicology

The belief that natural treatments are safer is not always true, therefore, it has been decided to evaluate the toxicity of medicinal plants, to guarantee greater safety for the creation of new drugs (Krishna and Sundararajan, 2020). Some studies on toxicology that have been carried out on *B. glabra* are presented below (Table 4).

**TABLE 4:** Toxicology of *B. glabra*.

Extract	Animals	Test	Dose	Result	Reference
Methanolic (plant organ used is not specified)	Wistar rats	Acute and subchronic toxicity	Acute: 2000 mg/kg Subchronic: 250, 500 and 1000 mg/kg	Acute: does not cause death or symptoms. Subchronic: do not generate significant changes.	Krishna and Sundararajan (2020)
Aqueous three-color bract	Zebra fish	Acute toxicity and teratogenic effect	0.3, 1, 3, 10, 30, 100 and 300 μg/mL	Acute: Pink extract with 85.51 μg/mL of 50% lethal concentration. Teratogenic: 20% edema of the yolk sac with dark pink extract, and purple bract hypopigmentation.	Teh et al. (2019)
Methanol and dichloromethane (DCM) from flowers	MDA-MB-231, MCF-7, CaSKi, DU-145, SW-480 cell lines	Cytotoxicity	500–15.625 μg/mL	IC_50_ (µg/mL)	Saleem et al. (2019)
				MDA-MB-231 = Methanol: 300.6	
				DCM: >500	
				MCF-7=	
				Methanol: 105.7	
				DCM: >500	
				CaSKi =	
				Methanol: 88.49	
				DCM: 180.1	
				DU-145 =	
				Methanol: 129.9	
				DCM: 180.9	
				SW-480 =	
				Methanol: >500	
				DCM: 304.7	
Ethanolic from bracts	Vero cell line and WRL-68	Cytotoxicity	NA	Vero: 269.10 ± 70.16 μg/mL WRL-68: 135.46 ± 20.43 μg/mL	Shalini et al. (2018)

To evaluate the cytotoxicity of the ethanolic extract of *B. glabra* bracts, they were exposed for 72 h with fetal human liver cells (WRL-68) and African green monkey (Vero) kidney cells, resulting in a mean inhibition concentration (IC_50_) of 269.10 ± 70.16 μg/mL for VERO cells and 135.46 ± 20.43 μg/mL for WRL-68 cells, considered the extract without toxicity since it did not exceed the negative control (Shalini et al., 2018).

To evaluate the acute toxicity and the teratogenic effect of the aqueous extract of three colors of bracts (purple, pink and strong pink) of *B. glabra*, zebrafish embryos were used. Acute toxicity was evaluated with the following concentrations: 0.3, 1, 3, 10, 30, 100 and 300 μg/mL, the pink bract extract being toxic to embryos with 85.51 μg/mL of 50% lethal concentration. The three extracts caused yolk sac edema as teratogenic results, highlighting a greater growth (20%) with the dark pink bract; in addition to hypopigmentation, which was observed to a greater extent with the purple extract. Despite this, the extract is not considered toxic since the embryos did not undergo major modifications (Teh et al., 2019).

The cytotoxicity of methanolic and dichloromethane extracts from *B. glabra* flowers was evaluated against different cancer cell lines, such as breast cancer (MDA-MB-231, MCF-7), cervical cancer (CaSKi), prostate (DU-145) and colon cancer (SW-480), resulting in the methanolic extract with the highest activity against the CasKi line, while the dichloromethane extract presented moderate activity (Saleem et al., 2019). The presence of certain phenolic compounds has been reported to cause apoptosis in cancer cell lines, this may be due to the polarity of the compounds (Yerlikaya et al., 2017).

The acute and subchronic toxicity of methanolic extracts of *B. glabra* was evaluated in albino Wistar rats, which were subjected for 90 days to a dose of 250, 500 and 1000 mg/kg for the subchronic test. At the end of this period, the animals were sacrificed, and hematological, biochemical, and histopathological parameters were evaluated, which resulted without significant variations compared to the control. For the acute toxicity test, 2000 mg/kg of extract was administered for 14 days, and no mortality or changes in respiratory symptoms, piloerection, tearing, or locomotor symptoms were recorded (Krishna and Sundararajan, 2020).

Toxicological tests offer us a different perspective since it is commonly believed that plant extracts using solvents such as methanol can cause some damage to the organism, but results such as Krishna and Sundararajan (Krishna and Sundararajan, 2020), it reminds us that the appropriate solvent can extract a greater amount of phytochemicals from the plant, which will provide better therapeutic properties.

With previous studies we can consider that the toxicity of *B. glabra* is null, this may be because the main compound present in the involucre is betalains, which have reported minimal toxicity and side effects (Sadowska and Bartosz, 2021). However, it is necessary to increase the number of *in vitro* and *in vivo* studies of this plant to provide greater security. In addition, it is important to add evidence on the toxicological effect of *B. glabra* in aquatic environments since bioassays using algae and invertebrates are extremely important to know the trophic impact of a substance in an ecosystem (Cangiano et al., 2002).

## Conclusion and future perspective

Ethnobotany is a tool that has allowed the search and choice of medicinal plants that are a novel alternative for the treatment of infections. Due to the current increase in microbial resistance to antibiotics, medicinal plants represent the largest reserve of phytochemical compounds available to counteract this problem; but currently this reserve needs numerous studies to correctly identify the secondary metabolites, as well as their mechanisms of action on the inhibition of bacterial growth.

The phytochemical profile of the involucre of *B. glabra* contain a variety of compounds, mainly betalains and phenols, providing a new opportunity to study their potential as antimicrobial agents and antibiofilm, but currently, there are no studies that demonstrate the values of minimum inhibitory concentration (MIC), minimum bactericidal concentration (MBC) and half maximal inhibitory concentration (IC_50_) necessary to validate the antibacterial activity with adequate concentrations to provide safety when using it in the health sector.

Current research should not focus only on the antimicrobial activity of the extracts, but on their antibiofilm activity, since this adherence gives them greater resistance to antibiotics and there are no drugs that specifically target this infection mechanism. Therefore, the potential of *B. glabra* as an antibiofilm should be investigated since it has an action on planktonic bacteria.

At the same time, taking advantage of the use of scanning electron microscopy is an option that would allow us to know how the phytochemistry of *B. glabra* extracts affects the structure of bacterial cell morphology and biofilms.

The use of *B. glabra* as a therapeutic agent based on traditional medicine still presents different challenges, first, to know its potential it is necessary to carry out a correct identification of the plant organ that allows to identify the diversity of secondary metabolites present, to enhance its therapeutic properties.

Finally, it is important to highlight the potential of this ornamental plant, not only to beautify landscapes but also its role in mitigating another current problem, which is air pollution in large cities.

We are invited to continue studying all those plants used in traditional medicine.
