# Supplementary material for: Bougainvillea glabra Choisy (Nyctinaginacea): review of phytochemistry and antimicrobial potential
Source: Front Chem. 2023 Oct 19;11:1276514. doi: 10.3389/fchem.2023.1276514 (PMC10620508; doi:10.3389/fchem.2023.1276514)
Supplement: Supplementary file 1 [file Table1.docx]

Supplementary Material

*Bougainvillea glabra* Choisy (Nyctinaginacea): Review of Phytochemistry and antimicrobial potential

Ingrid G. Ornelas García^1^, Alma L. Guerrero Barrera^1*^, Francisco J. Avelar González^2^, Norma A. Chávez Vela^3^. Daniela Gutiérrez Montiel^1^.

1. Laboratorio de Biología Celular y Tisular, Universidad Autónoma de Aguascalientes (UAA), Centro de Ciencias Básicas, Departamento de Morfología, Aguascalientes, México.
2. Laboratorio de Estudios Ambientales, Universidad Autónoma de Aguascalientes (UAA), Centro de Ciencias Básicas, Departamento de Fisiología y Farmacología, Aguascalientes, México.
3. Laboratorio de Biotecnología. Centro de Ciencias Básicas, Departamento Ingeniería Bioquímica, Aguascalientes, México.

*** Correspondence:**

Dra. Alma Lilian Guerrero Barrera. Laboratorio de Biología Celular y Tisular, Edificio 203. Universidad Autónoma de Aguascalientes, Centro de Ciencias Básicas, Departamento de Morfología, Av. Universidad 940. C.U. Aguascalientes, México. C.P.20100

Table S1: Phytochemistry isolated from bougainvillea involucre and antimicrobial properties.

| **Active principle** | **Compound name, CAS number and structure** | **Antimicrobial properties** | **Reference** |
| --- | --- | --- | --- |
| Betalains | Bougainvillein-V (15S-betanidin 6-0-sophoroside) (= [2’-O-β- glucoside)  Gomphrenin I (15S-betanidin 6-O-β-glucoside)  15S-Betanidine 6-O(6′′-O-E-4-coumaroyl)-β-sophoroside  15S-Betanidine 6- O (6′- O -E-4-coumaroyl)- β – sophoroside  15S-Betanidin 6-O(6’-O-E-4-Coumaroyl)-β-sophoroside  15S-Betanidin 6-O{2”-O-β-sophorosyl[6‘-O-E-caffeoyl) -(6”-O-E-4-coumaroyl)}-β-sophoroside  15S-Betanidin 6-O-{2’-O-β-glucosyl)[(6’-O-E-caffeoyl)-(6”-O-E-4-coumaroyl)]}- β-sophoroside  15S-Betanidin 6-O-[(2’-O-β-glucosyl)(6’,6”-di-O-E-4-coumaroyl)]-β-sophoroside  15S-Betanidin 6-O(6’,6”-di-O-E-4-coumaroyl)-β-sophoroside | **Betacyanins**  *S. aureus* and *B. cereus*  MIC: 0.75 and 0.5 mg/mL  *E. faecalis, B. cereus, Listeria monocytogenes* and *S. aureus*  MIC: 15.6 μg/mL  E. faecium  MIC: 0.78 mg/mL | Heuer et al., 1994  Wijesinghe and Choo, 2022 |
| Triterpenoid | Momordin IIc (quinoside D) [β-D-glucopyranosyl 3-O-[β-D-xylopyranosyl-(1→3)-O-(β-D-glucopyranosyluronic acid)] oleanolate] |  | Simon et al., 2006  Nguyen and Bhattacharya, 2022 |
| Flavonoids | Quercetin 3-O-α-L-(rhamnopyranosyl)(1→6)-[α-L-rhamnopy-ranosyl(1→2)]-β-D-galactopyranoside  Derivate quercetin 3-O-α-L-(4-caffeoylrhamnopyranosyl) (1→6)-[α-L rhamnopyranosyl (1→2)]- β-D-galactopyranoside | **Quercetin and derivatives**  *S. mutans*  MIC: 500 µg/mL  *S. aureus and P. aeruginosa*  MIC: 20 mcg/mL  *Micrococcus luteus* and *Shigella sonei*  MIC: 25 mcg/mL |  |
| Betacyanins | Betanidin-6-O-[(2”-O-β-sophorosyl)-(6´-trans-feruloyl -6”-O-trans-coumaroyl)]-β-sophoroside  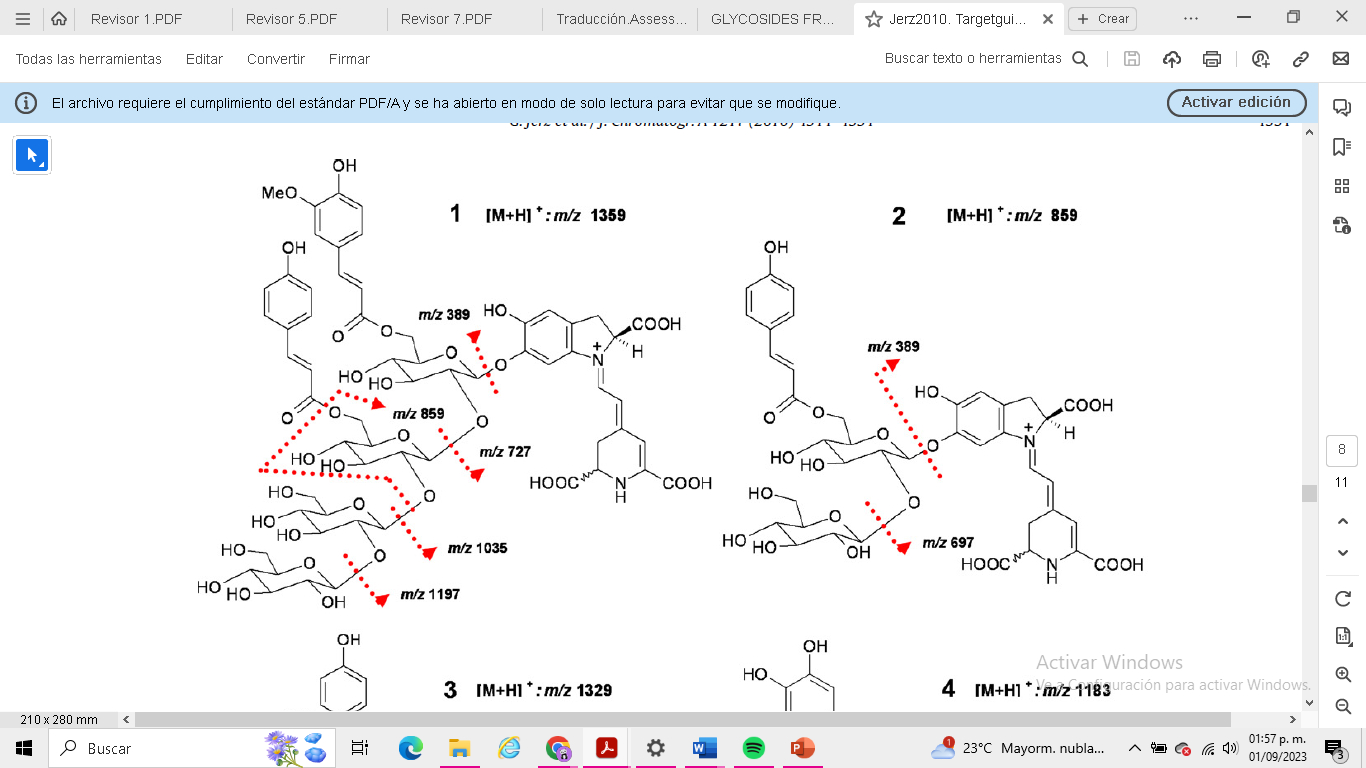  Betanidin-6-O-(6´-O-trans-4-coumaroyl)-β-sophoroside  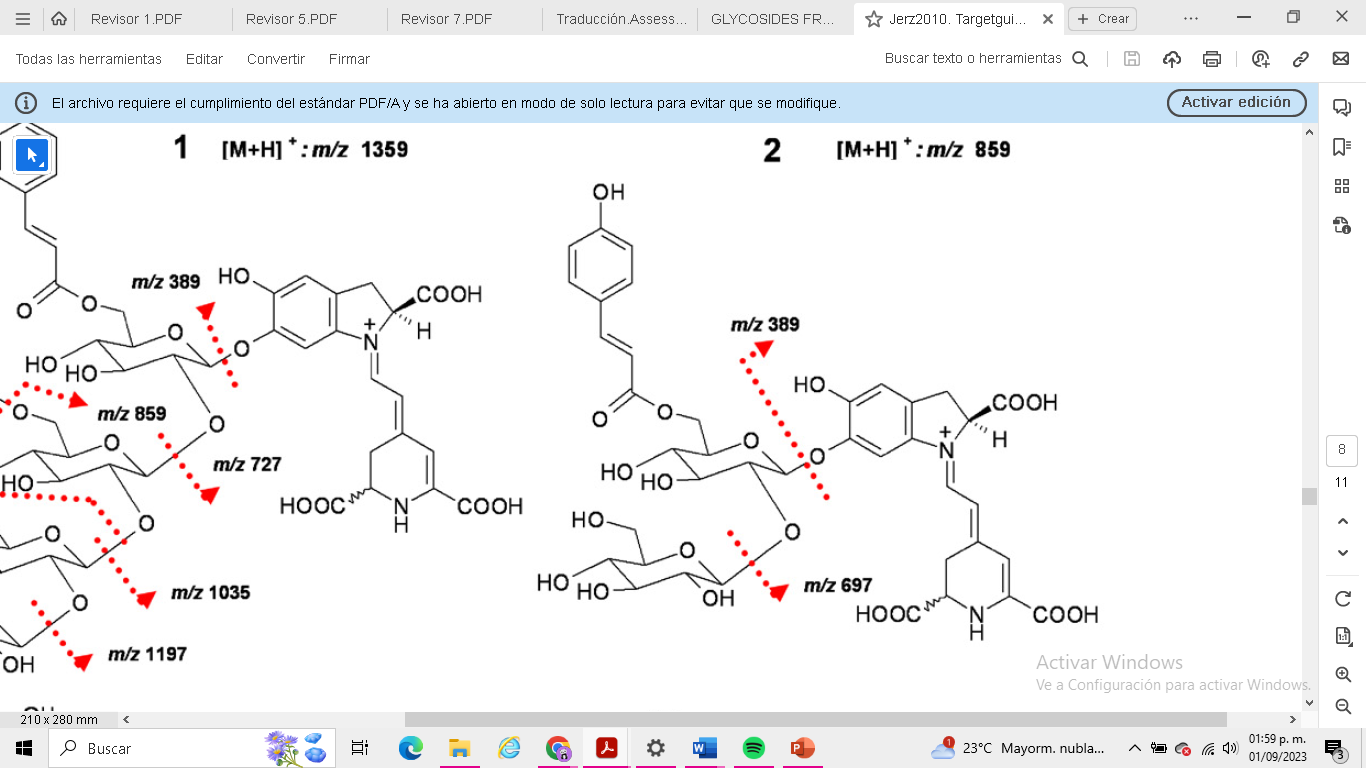  Betanidin-6-O-[(2”-O-β-sophorosyl)-(6´,6”-di-O-trans-coumaroyl)]-β-sophoroside  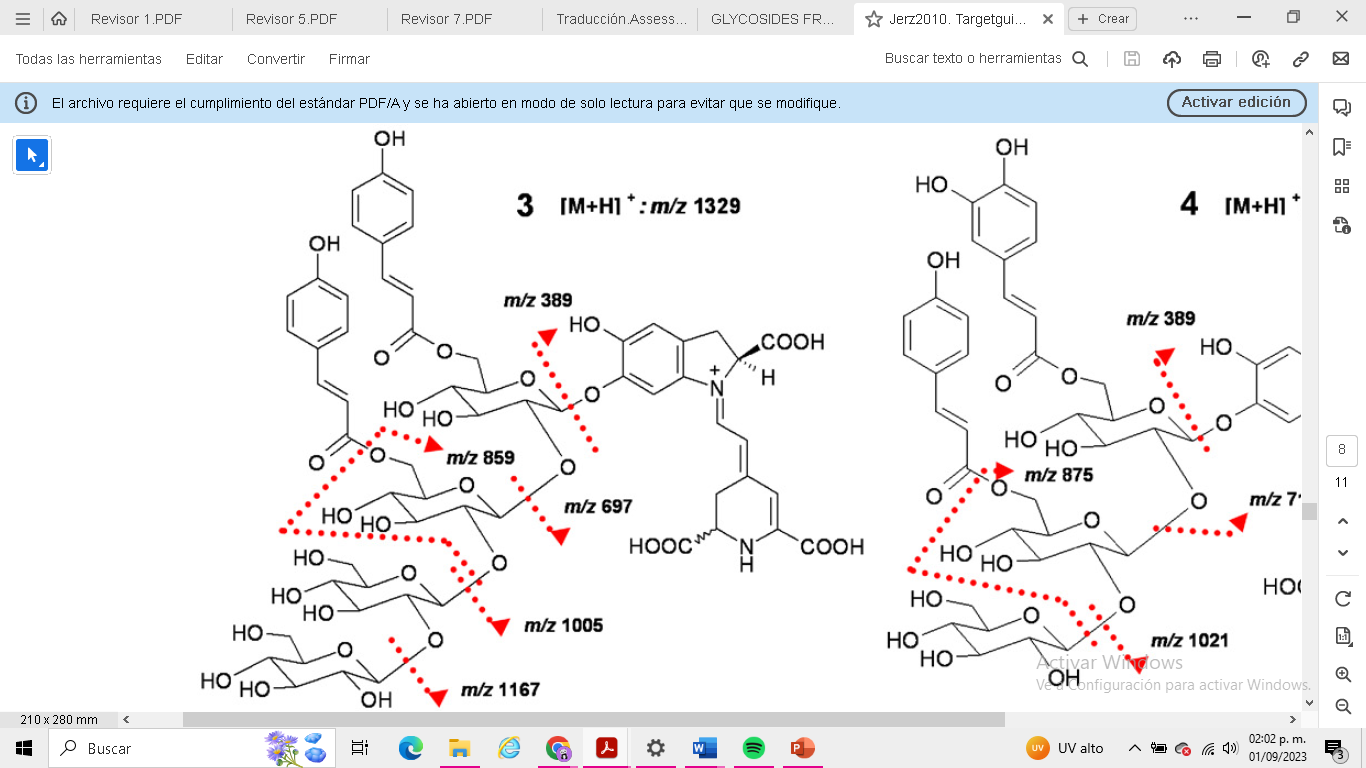  Betanidin-6-O-[(2”-O-β-glucosyl)-(6´-O-trans-caffeoyl-6”-O-trans-coumaroyl)]- β-sophoroside  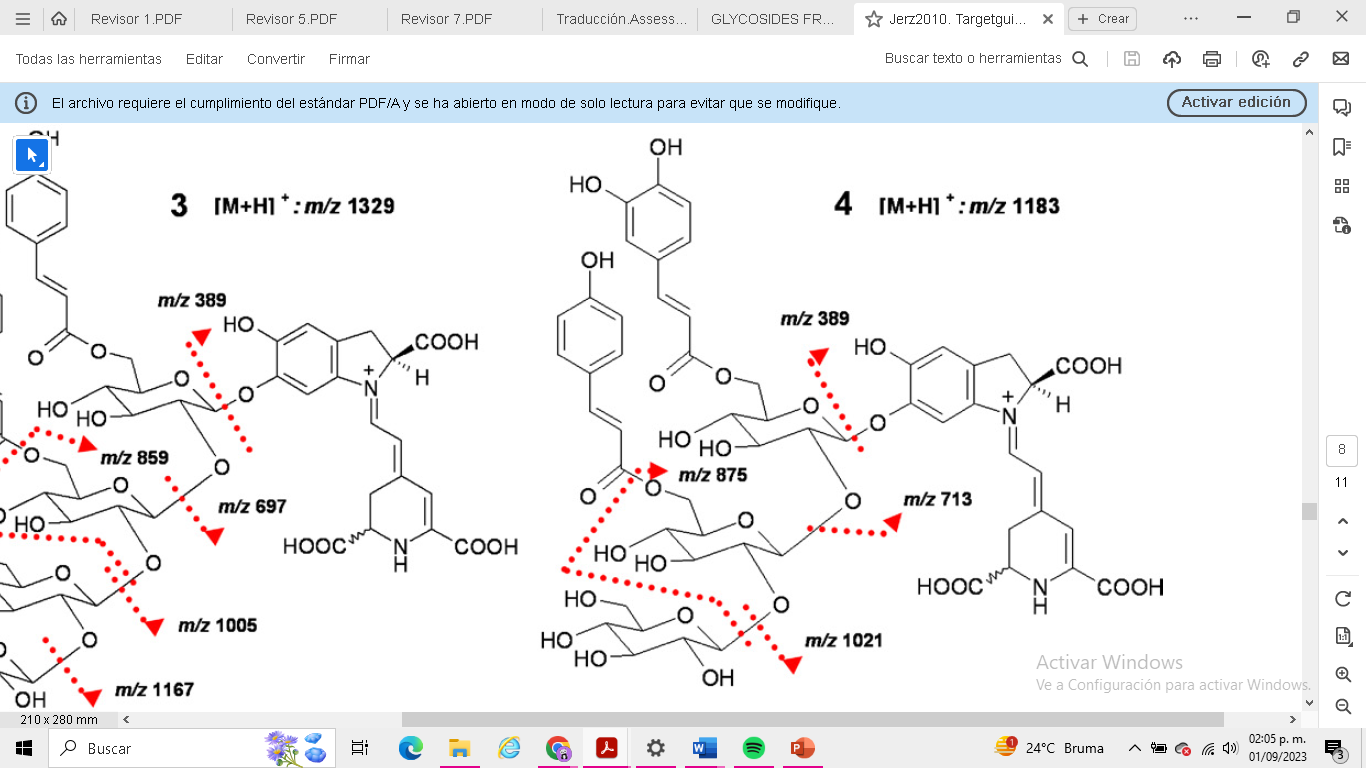  Betanidin-6-O-[(2”-O-β-glucosyl) -(6´-O-trans-coumaroyl-6”-O-trans-feruloyl)]-β-sophoroside  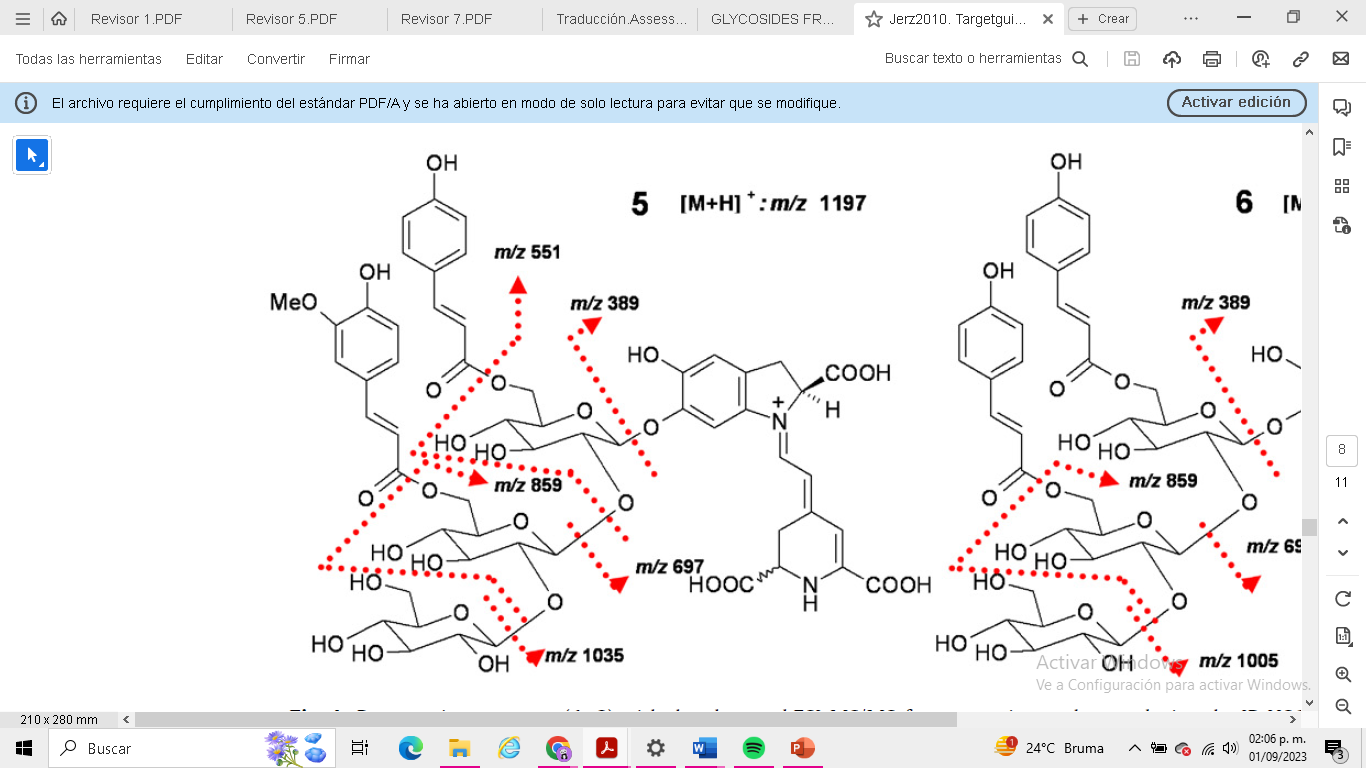  Betanidin-6-O-[(2”-O-β-glucosyl)-(6´,6”-di-O-trans-coumaroyl)]-β-sophoroside  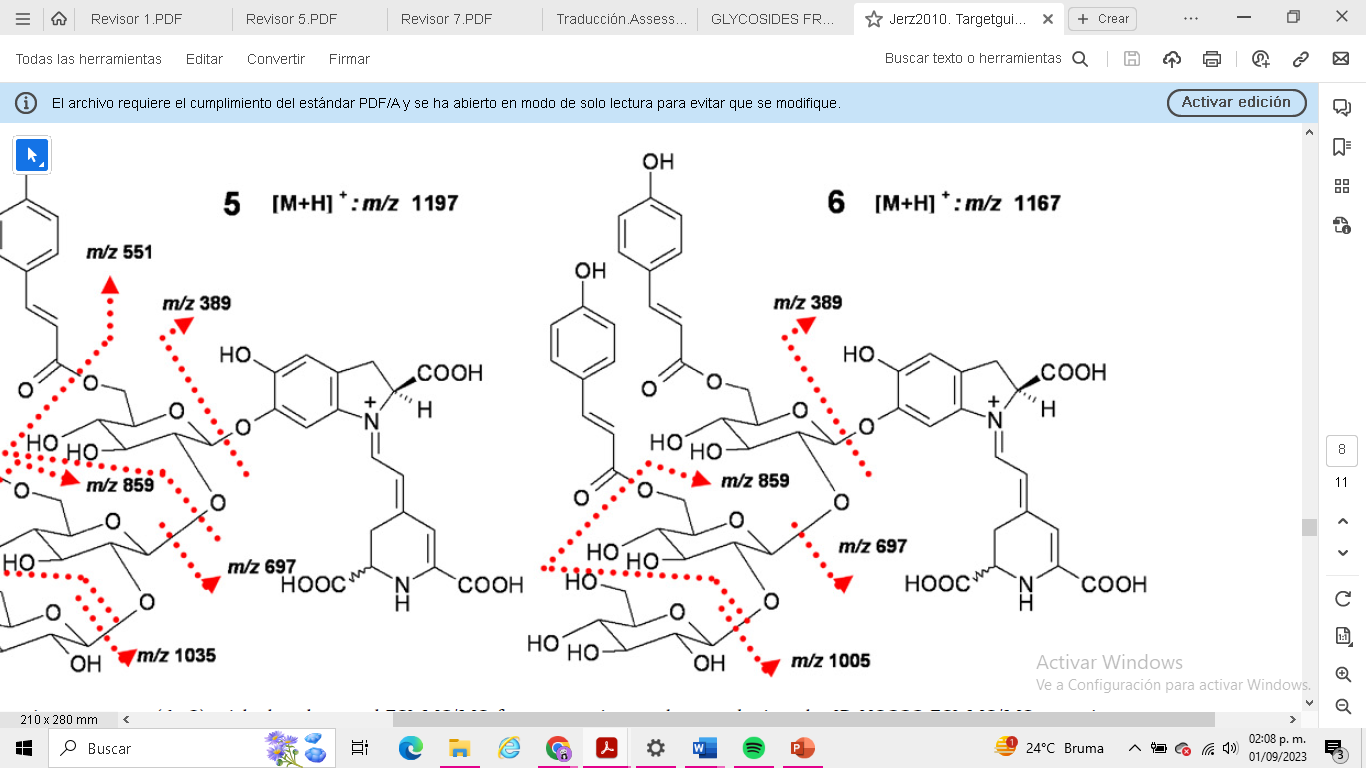 | **Betacyanins**  *S. aureus*  MIC: 3130 μg/mL  E. coli and P. aeruginosa  MIC: 1.5 mg/mL and 4.5 mg/mL  K. pneumoniae  MIC: 1.56 mg/mL  Aspergillus flavus  Botrytis cinerea  Cladosporium herbarum and  Fusarium oxysporum  MIC: 500 μg/mL  Candida albicans  MIC: 250 μg/mL  Rhizoctonia solani  MIC: 125 μg/mL | Jerz et al., 2010.  Wijesinghe and Choo, 2022. |
| Phenolic acids | Caffeic acid (331-39-5)  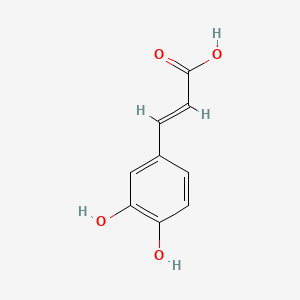  Chlorogenic acid (327-97-9)  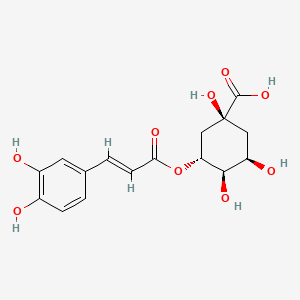  Ferulic acid (537-98-4)  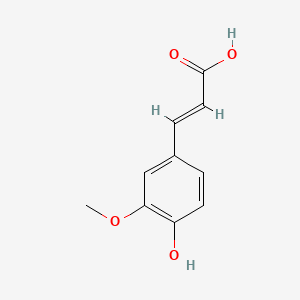  Gallic acid (149-91-7)  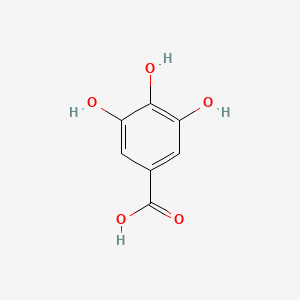  *p*-coumaric acid (501-98-4)  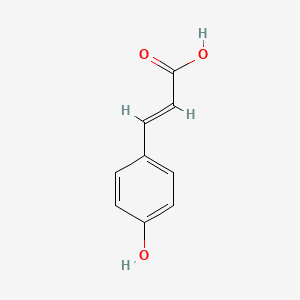  p-hydroxybenzoic acid (99-96-7)  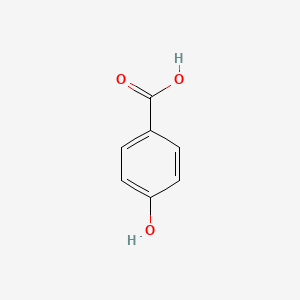  Protocatechuic acid (99-50-3)  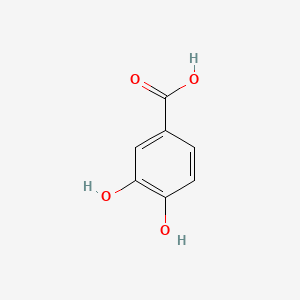  Sinapic acid (530-59-6)  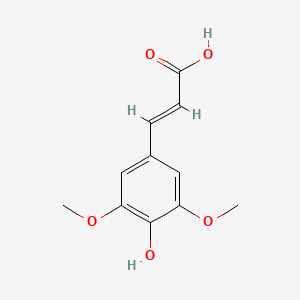  Syringic acid (530-57-4)  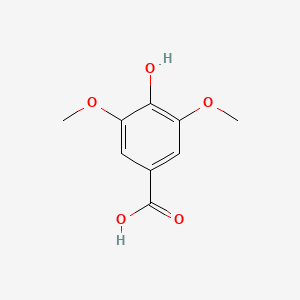  Vanillic acid (121-34-6)  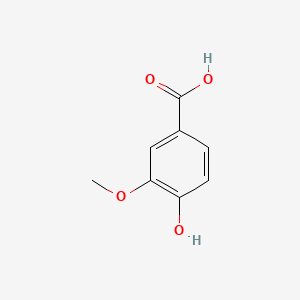 | **Caffeic acid**  *S. epidermidis*  MIC and MBC: 0.625 mg/mL  *S. aureus*  MIC and MBC: 0.625 and 1.25 mg/mL  *K. pneumoniae*  MIC and MBC: 5 mg/mL  *Citrobacter freundii*  IC_50_: 200-400 mg/mL  **Chlorogenic acid**  *S.  pneumoniae*  *S. dysenteriae*  MIC: 20 μg/mL  *S. aureus*  *B. subtilis*  *S.* Typhimurium  MIC: 40 μg/mL  *E. coli*  MIC: 80 μg/mL  **Ferulic and Gallic acid**  *P. aeruginosa*  MIC: 500 μg/mL  *E. coli*  MIC: 1500 μg/mL  *S.aureus*  MIC: 1750 μg/mL  *L. monocytogenes*  MIC: 2000 μg/mL  **p-coumaric acid**  *S. pneumoniae*  *S. aureus*  *B. subtilis*  *S. typhimurium*  MIC: 20 μg/mL  *E. coli*  MIC: 80 μg/mL  *S. dysenteriae*  MIC: 10 μg/mL  **p-hydroxybenzoic acid**  *Lactobacillus plantarum*  IC_50_: 165 μg/mL  *P. syringae*  IC_50_: 246-268 μg/mL  *Xanthomonas campestris*  IC_50_: 136 μg/mL  **Sinapic acid**  *B. subtilis*  MIC: 300 μg/mL  *E. coli*  MIC: 700 μg/mL  *S. aureus*  MIC: 300 μg/mL  *L. monocytogenes*  MIC: 200 μg/mL  *P. fluerescens*  MIC: 600 μg/mL  **Syringic acid**  *Cronobacter sakazakii*  MIC: 5000 μg/mL  **Vanillic acid**  *Enterobacter cloacae*  MIC: 600 μg/mL | Borges et al., 2013.  Cho et al., 1998.  Kaisoon et al., 2012.  Khan et al., 2021.  Lou et al., 2011.  Lou et al., 2012.  PubChem Compound Summary, 2023.  Niciforović and Abramovič, 2013.  Periferakis et al., 2022.  Qian et al., 2019.  Shi et al., 2016.  Wang et al., 2019. |
| Flavonoid | Apigenin (520-36-5)  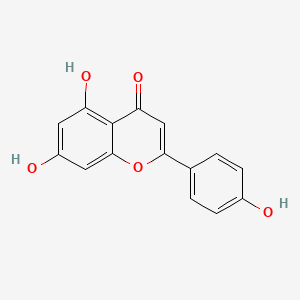  Kaempferol (520-18-3)  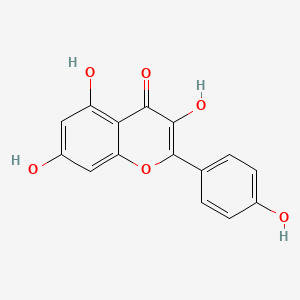  Myricetin (529-44-2)  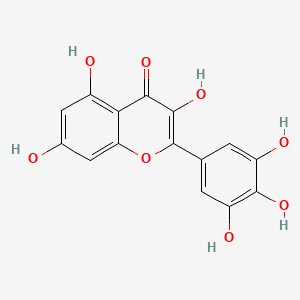  Quercetin (117-39-5)  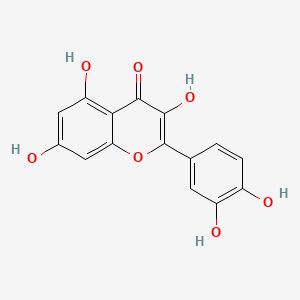  Rutin (153-18-4)  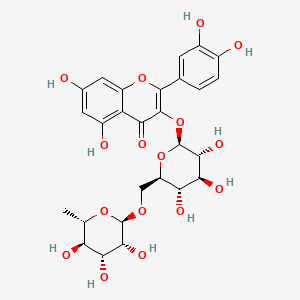 | **Apigenin**  *A. baumannii*  MIC: 2 and 64 µg/mL  *B. subtilis*  MIC: 8 and 16 µg/mL  *E. aerogenes* and *E. cloacae*  MIC: 64 µg/mL  *E. faecalis and K. pneumoniae*  MIC: 8 and 128 µg/mL  *E. coli*  MIC: 135.12, 128, 4 µg/mL  *Helicobacter pylori*  MIC: 25µg/mL  **Kaempferol**  *A. baumannii*  MIC: 8 µg/mL and 400 µg/mL  *E. coli*  MIC: 2 µg/mL, 13.3 µg/mL and 62.5 µg/mL  *K. pneumoniae*  MIC: 2 µg/mL, 4 µg/mL and 400 µg/mL  *Porphyromonas gingivalis*  MIC: 20 µg/mL |  |
| Flavonoid | Quercetin (117-39-5) |  | PubChem Compound Summary, 2023.  Shalini et al., 2018 |
| Flavonoids | 6-C-Rhamnopyranosylrhamnetin 3-O-glucopyranoside  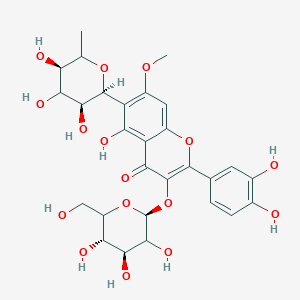  6-Hydroxyluteolin 5-rhamnoside  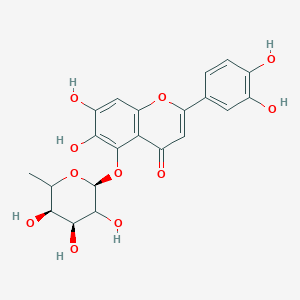  6-Methoxykaempferol 3-rhamnoside-7-(4'''-acetylrhamnoside)  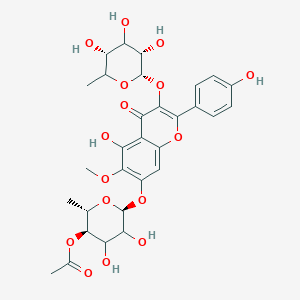  Brassicoside (17331-29-2)  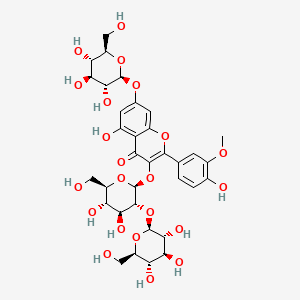  Egonol gentiobioside  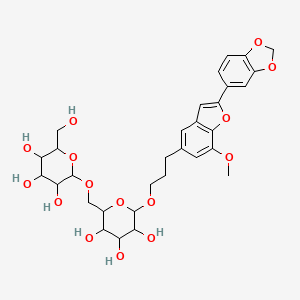  Isorhamnetin 3-glucosyl-(1->2)-[rhamnosyl-(1->6)-galactoside]  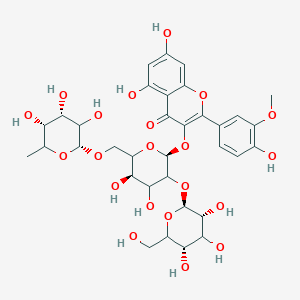  Isorhamnetin 3-glucosyl-(1->6)-galactoside  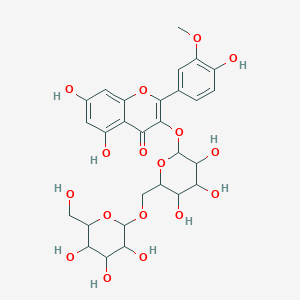  Isorhamnetin 3-rhamnosyl-(1->2)-gentiobiosyl-(1->6)-glucoside  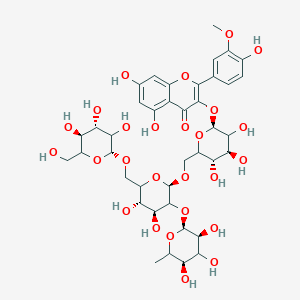  Isovitexin 2''-O-(6'''-(E)-p-coumaroyl)glucoside 4'-O-glucoside  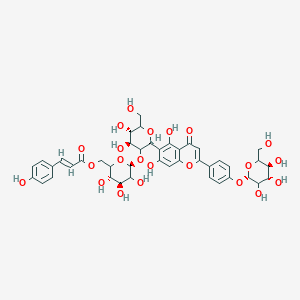  Isovitexin 7-(6'''-sinapoylglucoside) 4'-glucoside (212271-13-1)  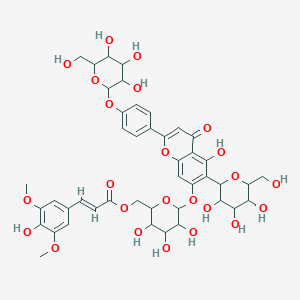  Kaempferol 3-(2G-glucosylrutinoside)  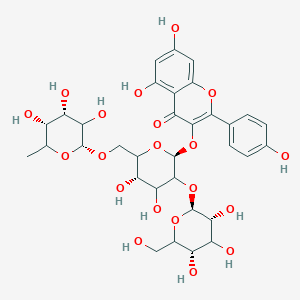  Kaempferol 3,4'-diglucoside-7-(2''-ferulylglucoside)  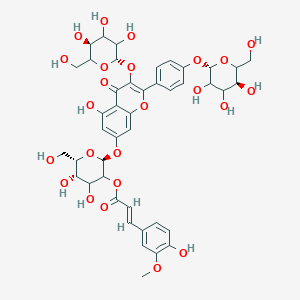  Kaempferol 3-neohesperidoside-7-(2''-ferulylglucoside)  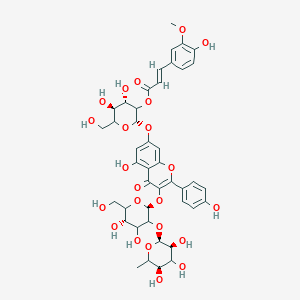  Kaempferol 3-rhamnoside-7-[6'''-ferulyglucosyl-(1->3)-rhamnoside]  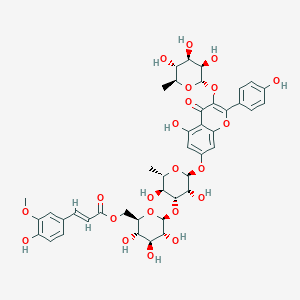  Laricitrin 3-rhamnoside  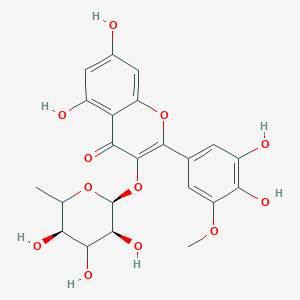  Luteolin 7-rhamnosyl (1->6) galactoside (20633-84-5)  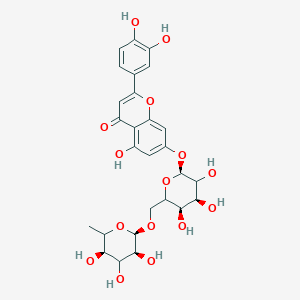  Robinetin 3-rutinoside  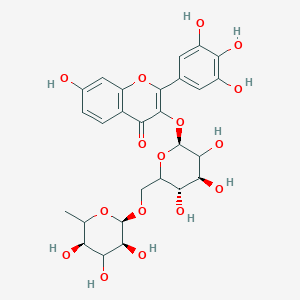  Robinin (301-19-9)  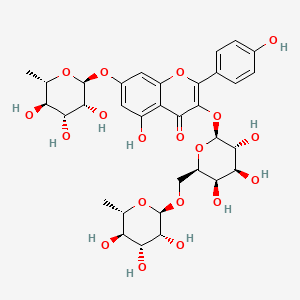  Tomentin 4'-glucoside  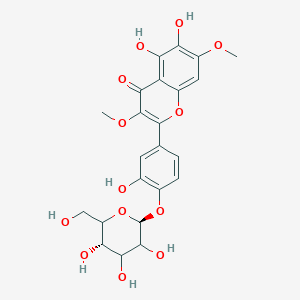  Tricetin 7-methyl ether 3'-glucoside-5'-rhamnoside  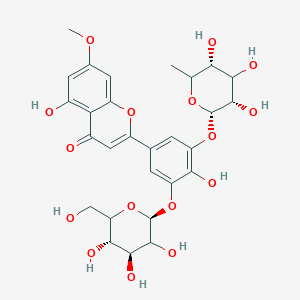  Viscumneoside III (118985-27-6)  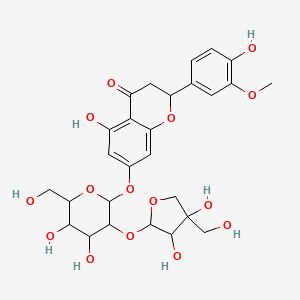  Vitisifuran A  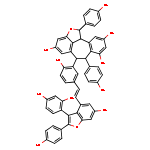 | **Kaempferol**  *S. typhi* and *S. typhimurium*  MIC: 12.5 µg/mL and 10.5 µg/mL  *S. aureus* and *S. epidermidis*  MIC: 125 µg/mL, 0.5 µg/mL, and 625 µg/mL  *Streptococcus pyogenes*  MIC: 6.25 µg/mL  B. subtilis and B. cereus  MIC: 6.25 µg/mL  *Mycobacterium bovis* and  *M. tuberculosis*  MIC: 62.25 µg/mL  *Proteus mirabilis* and *P. vulgaris*  MIC: 2 µg/mL, 300 µg/mL and 14.8 µg/mL  *P. aeruginosa*  MIC: 256 µg/mL and 10.2 µg/mL  *Prevotella intermedia*  MIC: 20 µg/mL  **Luteolin**  *E. coli*  MIC_50_: 67.25 µg/mL  *M. tuberculosis*  MIC: 25 µg/mL | Human Metabolome Database, 2023.  PubChem Compound Summary, 2023.  Periferakis et al., 2022.  Saleem et al., 2019.  Shamsudin et al., 2022. |
| Amino acids | *N*-Carboxyethyl-γ-aminobutyric acid (4386-03-2)  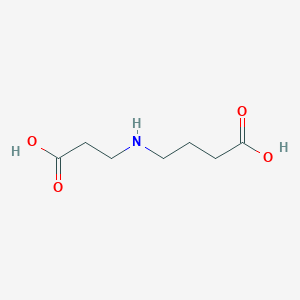  *N*-(1-Deoxy-1-fructosyl) phenylalanine (87251-83-0)  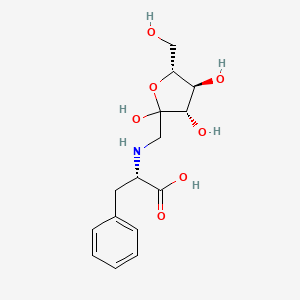 |  | PubChem Compound Summary, 2023.  Saleem et al., 2019.  Kim et al., 2015. |
| Glycoside | Lucuminic acid (190323-48-9)  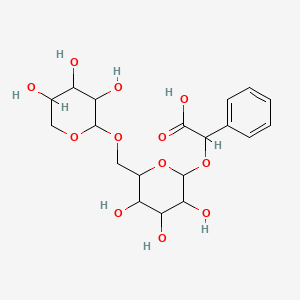 |  |  |
| Phenolic glycoside | Oenanthoside A  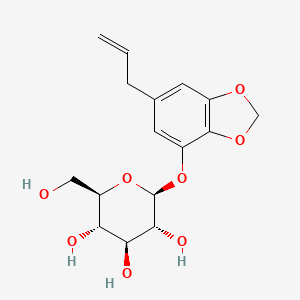 |  |  |
| Triterpenoid | Oleanolic acid 3-O-beta-D-glucosiduronic acid (26020-14-4)  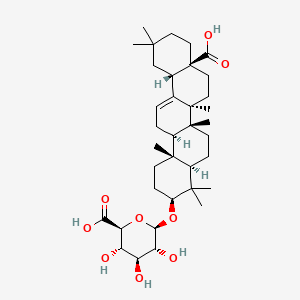 | **Oleanolic acid**  *L. monocytogenes*  MIC: 16-32 µg/mL  *E. faecium* and *E. faecalis*  MIC: 32-64 µg/mL |  |
| Flavonoid aglycones | Chrysoeriol methyl ether (491-71-4)  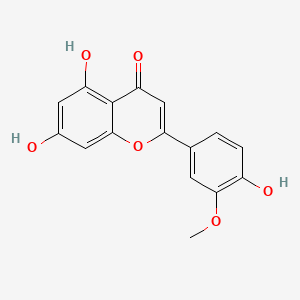  Myricetin derivate (529-44-2)  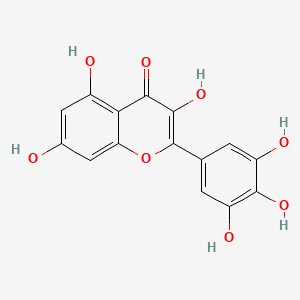  Quercetin (117-39-5)  Tetramethoxy flavone (1168-42-9)  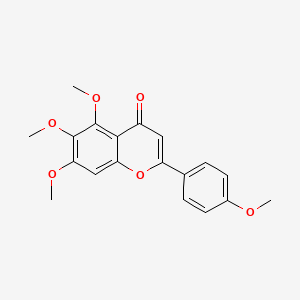 | **Myricetin**  *Mycobacterium tuberculosis*  MIC: 50 μg/mL  *E. coli*  MIC: 142 μg/mL  IC_50_: 1.18 mg/Ml  *P. aeruginosa*  MIC: 1.5 and 500 μg/ mL  **Tetramethoxy flavone**  *E. coli*  MIC_50_: 156.3 μg/ mL | PubChem Compound Summary, 2023.  El-Sayed et al., 2021.  Human Metabolome Database, 2023.  Shamsudin et al., 2022.  Taheri et al., 2020. |
| Phenolic acid derivatives | Caffeic acid derivative  Caffeic acid hexoside (14364-08-0)  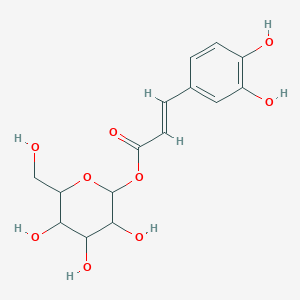  Caffeoylquinic acid (202650-88-2)  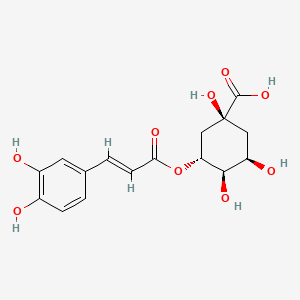  Coumaric-acid (501-98-4)  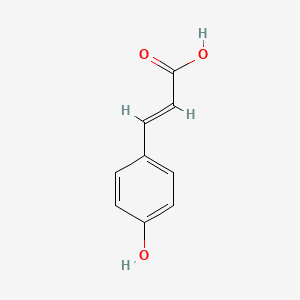  Ethyl gallate (831-61-8)  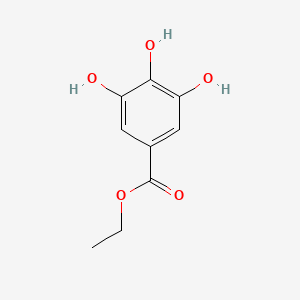  Ethyl gallate isomer  Ferulic acid derivative  Methoxy benzoic acid hexoside  Methoxycinnamic acid hexoside  p-hydroxybenzoic hexoside (99-96-7)  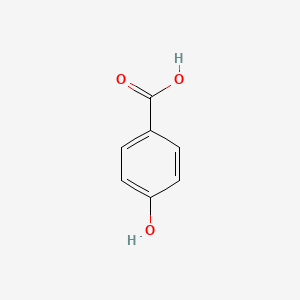  Protocatechualdehyde (139-85-5)  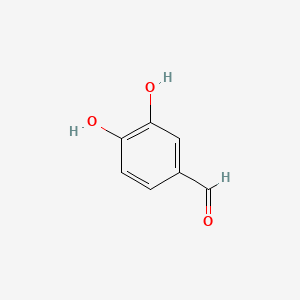  Sinapic acid-3-O-glucoside (117405-52-4)  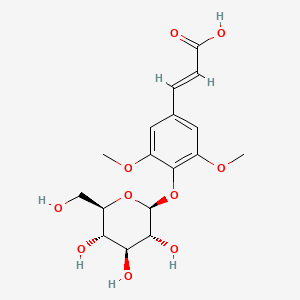  Sinapic acid-3-O-glucoside isomer  Trihydroxyursolic acid derivative | **Ethyl gallate fraction nine**  *P. aeruginosa, M. luteus* and *M. flavus*  MIC: 7.81 μg/mL  *P. vulgaris* and *S. aureus with* MIC: 15.62 μg/mL  **Protocatechualdehyde**  *Ralstonia solanacearum*  MIC: 40 μg/mL and 20 μg/mL | PubChem Compound Summary, 2023.  Li et al., 2016.  Bhat et al., 2023. |
| Free organic acids | Chebulic acid (23725-05-5)  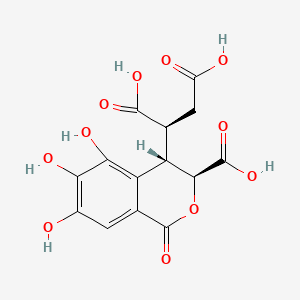  Ferulic acid (537-98-4)  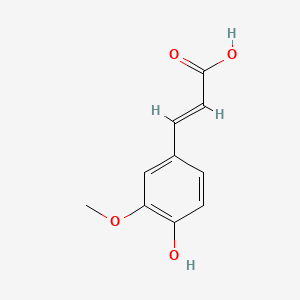  Ferulic acid isomer  p-hydroxybenzoic (99-96-7)  Salvianolic acid B (121521-90-2)  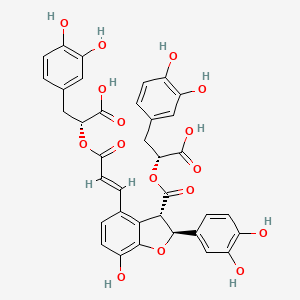  Syringic acid (530-57-4)  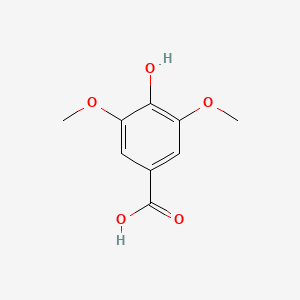  Syringic acid isomer | **Chebulic acid**  *R. solanacearum*  MIC: 26–52 μg/mL  *Neisseria gonorrhoeae*  MIC: 100 and 200 µg/mL | PubChem Compound Summary, 2023.  Dhingra et al., 2022. |
| Flavonoid-О-glycosides | Quercetin-*O*methacrylate  Acacetin-7-*O*-glucoside (4291-60-5)  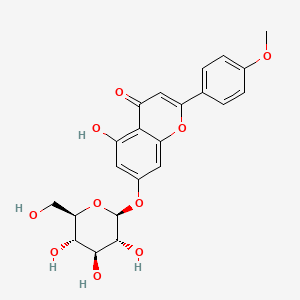  Liquirtigenin-7-*O*-glucoside  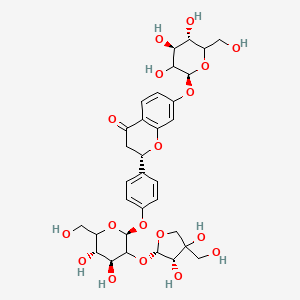 |  |  |
| Flavonoid glucuronides | Apigenin glucuronide (29741-09-1)  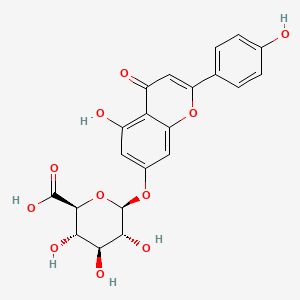  Luteolin-3'-*O*-(*O*-acetyl) *β-D-* glucuronide  Quercetin-3-*O*-glucuronide (miquelianin) (22688-79-5)  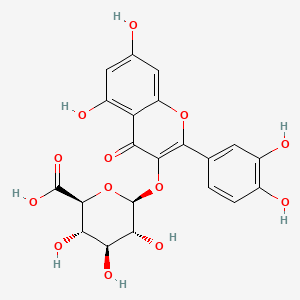 |  |  |
| Anthocyanins | Caffeoyl-glucose moieties  Cyanidin-3-*O*-acetyl- glucoside and isomers  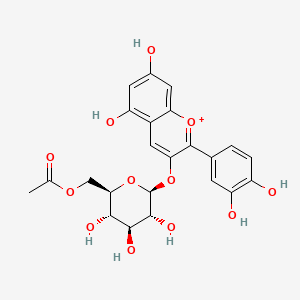  Delphinidin hexosyl pentosyl malonate and isomer  Guaiacyl pyrano malvidin-3-*O*-glucoside |  |  |
| Betacyanins | 5''-*O*- salicyl -2'-*O*-glucosyl betanin /isobetanin |  |  |
| Saponins | 3-*O*-glucuronide-29-hydroxyoleanolic acid  Chikusetsu saponins (CSs) Iva (3- *O- β- D-* glucuronopyranosyl-28-*O-β-D* glucopyranosyl oleanolic acid or 3-*O*-glucuronide oleanolic acid- 28-*O-*hexose and isomer) (26020-14-4)  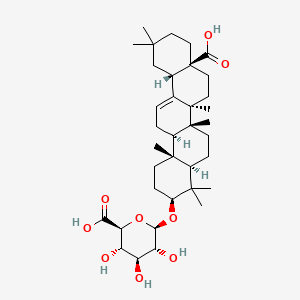 | **Chikusetsu saponins**  *E. coli* and *S. flexneri*  MIC: 16–32 μg/mL | PubChem Compound Summary, 2023.  Tagousop et al., 2018. |
| Hydrolysable tannins derivatives | Acetyl-*O*-galloyl-glucose  Hexahydroxydiphenoyl (HHDP) galloyl-glucose  Methyl trigalloyl glucosa  *O-*Galloyl arbutin (5991-00-4)  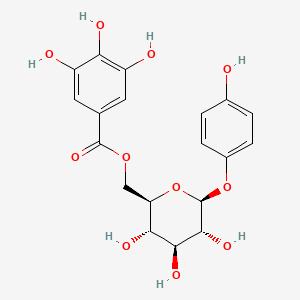  Trigalloyl hexoside (79886-49-0)  Trigalloyl levoglucosan |  |  |
| Cyclic tetrapyrrolic derivatives | 13^2^-hydroxypheophorbide -α-methyl ester and isomer (R=Me)  13^2^- hydroxypheophorbide-β- methyl ester (R=OCH) |  |  |
| Miscellaneous compounds | Fraxiresinol hexoside  Lanopalmitic acid (506-13-8)  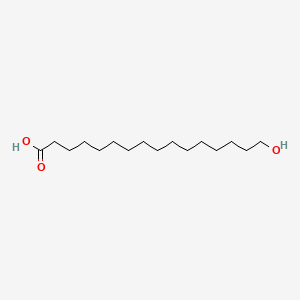  *N-*feruloyl tyramine and isomer (80510-09-4)  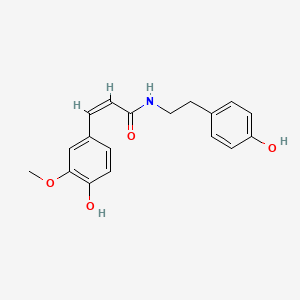  Sucrose (57-50-1)  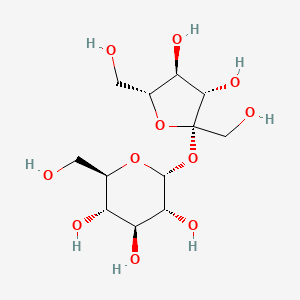 |  |  |
